# Educational strategies to improve health literacy for people with type 2 diabetes in low socio-economic communities: A realist review protocol

**DOI:** 10.1371/journal.pone.0352599

**Published:** 2026-07-30

**Authors:** Namalambo Mwenda - Ng’uni, Martin Heine, Fastone Goma, Monika Martens, Miriam Mapulanga - Lisulo, Brown Kamanga, Inonge Milupi, Susan Hanekom

**Affiliations:** 1 Department of Health and Rehabilitation Science, Stellenbosch University, Cape Town, South Africa; 2 Department of Physiotherapy, University Teaching Hospital, Ministry of Health, Lusaka, Zambia; 3 Julius Centre for Health Sciences and Primary Care, University Medical Centre Utrecht, Netherlands; 4 Institute of Sport and Exercise Medicine, Faculty of Medicine and Health Sciences, Stellenbosch University, Cape Town, South Africa; 5 Department of Physiological Sciences, University of Zambia, Lusaka, Zambia; 6 Department of Family Medicine and Population Health (FAMPOP), Faculty of Medicine and Health Sciences, University of Antwerp, Antwerp, Belgium; 7 Department of Physiotherapy, School of Health Sciences, University of Zambia, Lusaka, Zambia; 8 Endocrine Unit, Department of Internal Medicine, School of Medicine, University of Zambia, Lusaka, Zambia; 9 Department of Language and Social Sciences, School of Education, University of Zambia, Lusaka, Zambia; University of Diyala College of Medicine, IRAQ

## Abstract

**Introduction:**

Type 2 diabetes (T2D) has emerged among the top ten causes of disability and mortality worldwide. Health literacy is crucial for effective self-management to reduce the burden associated with T2D. Studies have reported the effectiveness of educational strategies for improving health literacy to promote good health and well-being. However, contextual factors influence the effectiveness of these strategies. Therefore, this paper intends to explain how and why context shapes the mechanisms through which educational strategies work to improve health literacy for adults with type 2 diabetes in low socioeconomic communities.

**Methods and analysis:**

Theory-driven realist review methods will explain how and why contexts activate different mechanisms through which educational strategies work to produce intended or unintended outcomes in low socioeconomic communities. The following five steps of realist review, which are non-linear and iterative, will be undertaken: (i) Define the scope of the review and locate existing theories on educational strategies to improve health literacy, (ii) Develop the initial programme theories, (iii) Search for evidence, (iv) Select papers and appraise, and (v) Extract data and synthesis. The following databases will be searched, but not limited to, PubMed, Education Resource Information Centre (ERIC), and PsycINFO. Papers will be selected based on relevance, richness, and rigour. Data extraction will follow both inductive and deductive approaches. The Intervention-Context-Actor-Mechanism Outcome (ICAMO) configurations will be utilised to analyse and synthesise data using retroductive reasoning. Finally, the revised programme theories, which explain how the educational strategies are expected to work across different contexts in low socioeconomic communities, will be prepared and disseminated.

**Conclusion:**

The findings may inform practice, influence policy, and contribute to the design of health literacy programmes in similar settings. **The realist review is registered with the Open Science Framework: (https://osf.io/9w867**)

## Introduction

The burden of type 2 diabetes (T2D) is increasing worldwide, becoming a major contributor to disability and mortality [1]. Low and middle-income countries (LMICs) bear approximately 80% of the global diabetes burden [2], with 77% of the unmet need for diabetes care [3]. Studies, including those from LMICS, have particularly highlighted poor self-management behaviours, poor treatment adherence, untreated diabetes, and late diagnosis as contributors to this burden among individuals with T2D [3–7]. Poorly managed diabetes increases the risk of tuberculosis [8] and worsens HIV/AIDS complications [9]. It similarly contributes to mental illnesses such as depression and anxiety, leading to poor health-related quality of life [10]. All these factors have social and economic implications, imposing substantial costs on already burdened healthcare systems, communities, and people with diabetes [11]. Reducing this burden depends on effective control of diabetes, which requires daily active self-management, a task influenced by health literacy [12].

Health literacy, the key enabler of self-management [12] is defined by the World Health Organisation (WHO) as *“the ability of individuals and communities to access, understand, appraise, remember, and apply health-related information in everyday life, continuously throughout the life course.*” [13]. Evidence indicates that health literacy is positively associated with improved access to and utilisation of the healthcare system [14]. Furthermore, it enhances patient-provider interaction, which strengthens individuals’ health-related knowledge and beliefs and enhances participation in decision-making regarding their health [14]. Educational strategies function as key mechanisms for improving health literacy, equipping patients with problem-solving skills that facilitate effective self-management [15,16].

However, the implementation and outcomes of educational strategies are influenced by the complex and dynamic nature of the social systems in which they are embedded [17]. Individual responses to educational strategies are shaped by a range of contextual factors that are within the broader social determinants of health [18]. These include socioeconomic status, cultural beliefs, community perception, existing relationships and hierarchies, and the state of the healthcare system [18–20]. These contextual factors significantly affect how knowledge and skills are acquired, interpreted, and applied. More specifically, low levels of education and poverty, which are prevalent in LMICs, may negatively impact health literacy [21,22]. Consequently, these factors affect health-seeking behaviours, processing of health education, engagement with health providers, adaptation to change, and adherence to treatment plans, leading to higher risks of complications, disability and mortality [21,23–25]. This multitude of contextual barriers constitutes a complex social system for the implementation of educational strategies to enhance health literacy.

In response to these contextual challenges, several studies have generally recommended person-centred approaches with multiple strategies as effective methods that could work in low socioeconomic communities [17,21,26–30]. Educational strategies advocated by many scholars include the use of role-play, discussions, and teach-back [17,26,30,31]. Other strategies include illustrated materials with texts that could be delivered in a group or at an individual level, depending on a person’s preferences and contextual needs [26,28,31]. Equally, modern technologies, including telecommunication and digital devices, have proven effective in certain contexts in improving medication adherence and self-management behaviours [27,32,33]. However, poor usability, digital illiteracy, and low rates of sustainability and accessibility have been reported, especially among elderly populations [32]. Hence, the importance of carefully considering context, content, and methods of communication to prevent misunderstandings, as these factors can lead to varying behavioural learning and health outcomes [34].

In addition to a person-centred and multi-strategy approach, the timing and place of information delivery are equally essential to improving health literacy. Some scholars recommend providing information at the time of diagnosis, as stressful events make the recipient more responsive to information to act [17]. Davies et. al recommend delivery of this information at four major time points: at diagnosis, annually or when not meeting the target, when complications set in, and when care transition occurs [28]. Other researchers recommend delivering health information in settings where it is most likely to be applied, within communities or healthcare settings, thereby reducing barriers to utilisation [15] and addressing situational demands and complexities experienced by patients [34]. Collectively, these studies acknowledge the importance of tailoring educational strategies to specific contexts to improve health literacy [17,26–29,32,34]. Ideally, educational strategies should be theory-tested and consider context to inform effective and sustainable health literacy [35,36].

Although these studies have evaluated educational strategies aimed at improving health literacy in people with T2D, their findings are not easily transferable to diverse contexts and circumstances [11,17,21,27,29,31,32,37,38]. Other studies on health literacy focused primarily on organisational health literacy within public health and healthcare settings [39,40]. Thus, creating a gap in patient-centred educational strategies that can be transferable and implementable within low socioeconomic communities. Building on prior research, this study employs a realist review methodology to examine educational strategies that improve health literacy among people with type 2 diabetes, with particular emphasis on disadvantaged populations.

The realist review is well suited, as it provides a causal explanation that enables transferability of strategies across different circumstances [41]. The assumption is that an educational strategy that works in a particular context, group, or for an individual may not work the same way in a different group or even the same person under different circumstances [42]. Therefore, this study aims to utilise a realist review approach to explain which educational strategies work (or do not work), for whom, why, and how, in what circumstances, to improve health literacy in adults with T2D in low socioeconomic communities. The specific objectives are to:

- Identify educational strategies that improve health literacy in individuals with T2D in low socioeconomic communities.
- Describe how contexts within low socioeconomic communities influence the mechanisms through which educational strategies work to improve health literacy.
- Explain why these contexts and which context conditions influence the mechanisms through which educational strategies work.

## Materials and methods

### Realist review team

**Fig 1:**
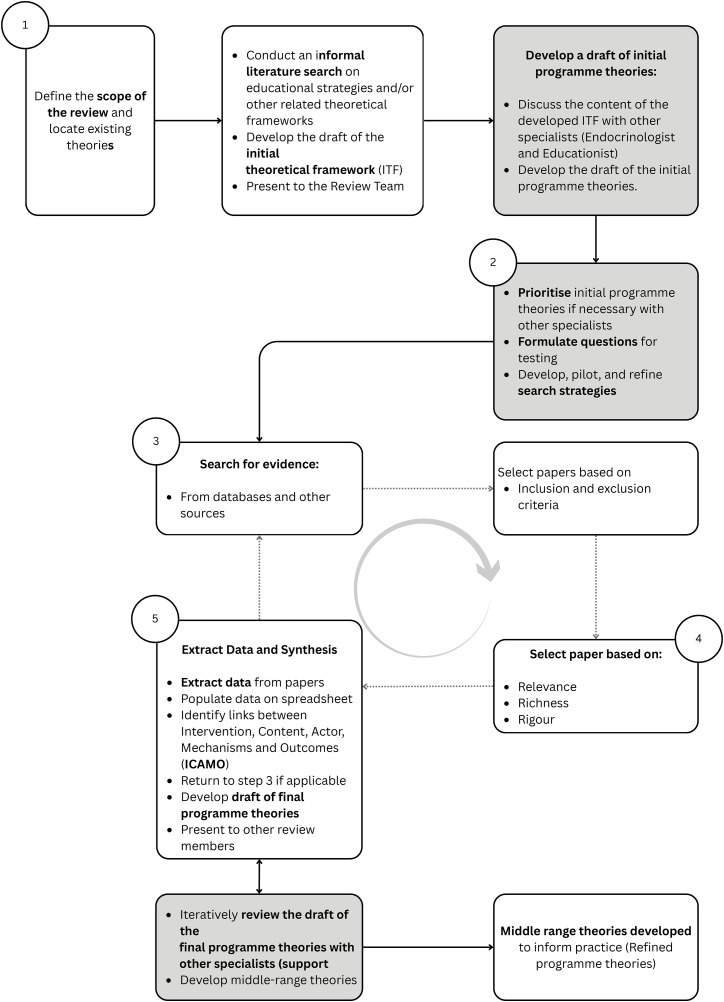
Realist review – (The light grey shading illustrates the joint work to be undertaken by the core team in collaboration with the support review team).

This study is conducted by a realist review team comprising a core team and a support team, all with expertise across various relevant fields. The core team consists of a rehabilitation specialist (SH), an implementation science expert (MH), a specialist in primary health care (FG), the principal investigator (NMN), and an assistant researcher (MML), all with experience in low-resource settings. Furthermore, the core team includes a realist methodology expert (MM), with expertise in global public health and implementation science. The core team remains actively engaged throughout the realist review as illustrated in Fig 1. The support team includes contextual experts, such as an education scientist (IM) and an endocrinologist (BK), recruited through the core team’s network. The educationist plays a crucial role in health literacy strategies [40] by providing expertise in identifying relevant learning theories and educational strategies that can be applied in diverse contexts to enhance health literacy. Meanwhile, the endocrinologist, as a specialist in diabetes, provides valuable insights into patients’ needs for managing the condition [43].

### Realist review

The realist review adopts a theory-driven methodology, appropriate for identifying educational strategies that work in diverse, complex, and dynamic social settings. Grounded in the realist philosophy of science, it seeks to explain causal relationships by examining the interaction between Context, Mechanisms, and Outcome, enabling transferability of strategies across contexts through the principle of generative causation [44,45]. Generative causation examines how specific mechanisms are activated in a particular context to produce intended and unintended outcomes [42].

The foundational analytic framework is the Context-Mechanism-Outcome configuration represented by CMOc. Context refers to the social or geographical conditions in which educational strategies are implemented [41]. Mechanism denotes underlying entities, processes, forces, and resources that generate change, often unobservable (e.g., human reasoning or social interaction) [41,46,47]. Outcomes in this study may be proximal, which include accessing, understanding, appraising, and applying health information, or distal, encompassing self-management behaviour and broader health outcomes [12].

To refine the analysis of educational strategies, two additional explanatory elements are incorporated into the CMO configuration: the ‘Intervention’ and the ‘Actors’ [48]. The intervention captures the components of the educational strategies (e.g., methods, modes, and media). The inclusion of Actors acknowledges people’s agency in the design and implementation of educational strategies. Actors include (e.g., people with diabetes, families, peers, educators, health providers) [49]. The Actors’ agency influences how health information is interpreted, influencing self-management outcomes. Therefore, this study employs the Intervention Context Actor Mechanism (ICAMO) configuration (framework). Definitions of the ICAMO elements are presented in Table 1.

**Table 1:** Definitions of Intervention, Context, Actors, Mechanisms, and Outcome (ICAMO) in this review.

Abbreviation	Definition
**I**	**Intervention** (educational strategies) encompasses the component/s of educational strategies (e.g., **Mode** (in person/virtual, group/individual), **Medium** (tools used – visual aids, leaflets, YouTube), **Methods** (teaching methods – discussion, Role play), **Place** (community/health centre), **timing** (stage of change/diagnosis), and **duration (length of education session/programme)** of delivery of health information)
**C**	**Context** is described as the circumstances and conditions that include place (urban/rural), time, socioeconomic status, rules, and sets of relationships within which a particular programme operates, which help explain the outcome (s), and in this realist study, specifically those conditions that influence mechanisms through which educational strategies work to improve health literacy.
**A**	**Actors** include those who are targeted by the educational strategies and those involved in the delivery (e.g., people with diabetes, family, peers, communities, educators, health care providers, organisations, and health care systems).
**M**	**Mechanisms** are defined as underlying individual, social, or system processes, forces, and/or powers, respectively (e.g., ranging from motivation and individual learning to social support, peer pressure, and peer learning; social norms getting activated and organisational (or wider system) learning surrounding health literacy) **that are triggered in specific contexts** through a programme **to give a particular outcome.**
**O**	**Outcomes** relate to proximal and distal outcomes of health literacy, which include understanding one’s health, health information, and the healthcare system; accessing, appraising and utilising health information; health-seeking; collaborating with health providers; good self-management behaviour; and health outcomes.

### Steps of the realist review

This review is informed by the five steps of a realist review, which are non-linear and iterative [44]. The reporting will be guided by RAMESES publication standards: realist synthesis to ensure methodological rigour and transparency (see S1 Table) [50]. The five steps that will be undertaken to conduct the realist review are as follows: -

- Step 1: Clarifying review scope and locating the existing theories,
- Step 2: Develop the initial programme theories
- Step 3: Search for evidence
- Step 4: Select and appraise evidence
- Step 5: Extract data and synthesis

An overview of the realist review process, adapted from Kantilal et al. [51], has been illustrated in Fig 1.

The five steps to follow in this review process are outlined below, and the first two have already been completed:

#### Step 1: Clarifying review scope and locating existing theories.

In realist review, theories inform the initial programme theories, providing a framework for synthesising evidence from empirical studies, programmes, stakeholders, experts, and literature. To locate existing theories, the core team conducted a purposeful literature search to identify frameworks for educational strategies to improve health literacy among individuals with type 2 diabetes in low socioeconomic communities. Low socioeconomic communities are characterised by high unemployment, unstable work, limited income, low educational attainment, and restricted access to resources [52]. The search focused on frameworks addressing health literacy and self-management in chronic diseases, as they offer accessible, theory-based explanations of causal pathways [46,47,51]. The integrated model of health literacy (IMHL) was selected as it aligns with the definition of health literacy and provides a socioecological lens for understanding behaviour change [14,53].

The IMHL highlights the multiple-level contextual factors (context) that influence the acquisition of knowledge, skills, and competences (mechanisms) to access, understand, appraise, and apply health information to promote self-management behaviours and good health outcomes (outcomes). The model comprises twelve components across the healthcare system, disease prevention, and health promotion. However, this study focuses on the health care domain at the individual level to address barriers to health literacy [54]. The model has been adapted from Sorensen et al. [14].

The IMHL acknowledges that self-management behaviour is influenced by interacting factors at personal, social, institutional, and societal levels largely beyond an individual’s control. Given the complexity of type 2 diabetes, comorbidities, and socioeconomic factors, a multiple-level framework was considered. However, addressing all levels is impractical, and focusing solely on the individual level is not effective and widens health inequities [47,55]. To balance effectiveness and feasibility, the IMHL retains two levels: the intrapersonal and the interpersonal levels, while also incorporating institutional interaction with the health providers.

Therefore, substantive theories from sociology, psychology, and education were purposively selected to inform strategies at these levels [56–61]. They were subsequently examined in applied studies within low socioeconomic communities to identify Outcomes, Mechanism, and Context [60–68]. These theories were consolidated by grouping similar theories and categorising them by their level of influence within the social system [62]. At the intrapersonal level. Patient Activation theory and Transtheoretical Model emphasise person-centred strategies, level of activation, and stage of change [59,63]. At the interpersonal level, Social Cognitive and Social Support theory highlights observational learning, personal agency, and modifying the external environment [64–67]. At an organisational level, Empowerment theory, rooted in Paulo Freire’s pedagogy, addresses structural factors, power dynamics, and active participation [57,68]. These theories were integrated into the IMHL to form the Rough Initial Programme Theories (IRPT) for further discussion with the support team in step 2.

#### Step 2: Develop initial programme theories.

This step prioritised and developed the initial programme theories for evidence testing and synthesis. Programme theories are defined as an abstract description of the components of the interventions and how they are expected to do or work to achieve a particular outcome, presented as an ICAMO framework in this study. The initial Rough Programme theory was circulated in written and diagrammatic form to the review team a month before a virtual meeting. The meeting included the core team and support team (SH, MM, NMN, IM, BK), who prioritised the theories and shaped the Initial programme Theories (IPT). Feedback from the support team members (IM and BK), who had not participated in developing the IRPT, confirmed that all four theories were essential and complementary, and that they addressed different stages of change and levels of social influence. Some educational strategies were later revised to clarify the causal pathway. The team agreed that the theories could be implemented in the low socioeconomic communities and should be taken forward for testing. Subsequently, the initial programme theory was developed, and the statement is as follows:

If educational strategies for people with type 2 diabetes in low socioeconomic communities incorporate person centred, structured practice, social support, and empowerment approaches **(C)**, **then,** they are more likely to access, understand, appraise, and utilise the health information and have good self-management behaviour and health outcomes **(O)** because they will have participated actively in the management of their condition, acquired the necessary knowledge, skills, support, and made informed decisions and set goals, which reduces diabetes related stress, increase motivation, problem solving skills, resources ultimately, self-efficacy, and ownership of their condition (M).

The intervention and actors’ component of the ICAMO will be integrated during the testing phase. The full statements of the associated hypotheses are presented in Table 2.

**Table 2:** Hypothetical statements for the initial programme theories.

Social-ecological level	Hypothetical statements
	Educational strategies aimed at improving health literacy among people with type 2 diabetes in low socioeconomic communities should: -
**Intrapersonal level**	**Transtheoretical Model**
	Be person-centred, tailored to an individual’s stage of behavioural change and specific needs to enhance decision-making and self-efficacy, thereby influencing lifestyle modification and sustained good self-management behaviour *(use pictograms, testimonies, videos, and printed booklets with easy-to-understand language (culturally appropriate)*
**Interpersonal level**	**Social cognitive theory**
	Promote structured practice and provide successful role models to improve self-efficacy (motivation and confidence) for effective self-management behaviour. (*Promote active participation, group therapy, and provide similar models, feedback, and goal setting) (Patients’ preferences to be upheld)*
	**Social support theory**
	Provide tangible (e.g., money, materials) and intangible (emotional and psychological support) support to reduce diabetes related distress through involvement of family, peers, and other caregivers for improved self-management behaviour *(Group therapy, incentives, involvement of peers and family; patients’ preferences to be upheld)*
**Organisational Level**	**Empowerment theory**
	Promote critical awareness of the internal and external environment, available resources, and encourage active participation and equal authority in managing their condition to increase autonomy, improve decision-making, self-management behaviour, and health outcomes. *(one-on-one, group therapy, interactive sessions, use of visuals, practical sessions, setting of goals, monitoring feedback)*

**Fig 2:**
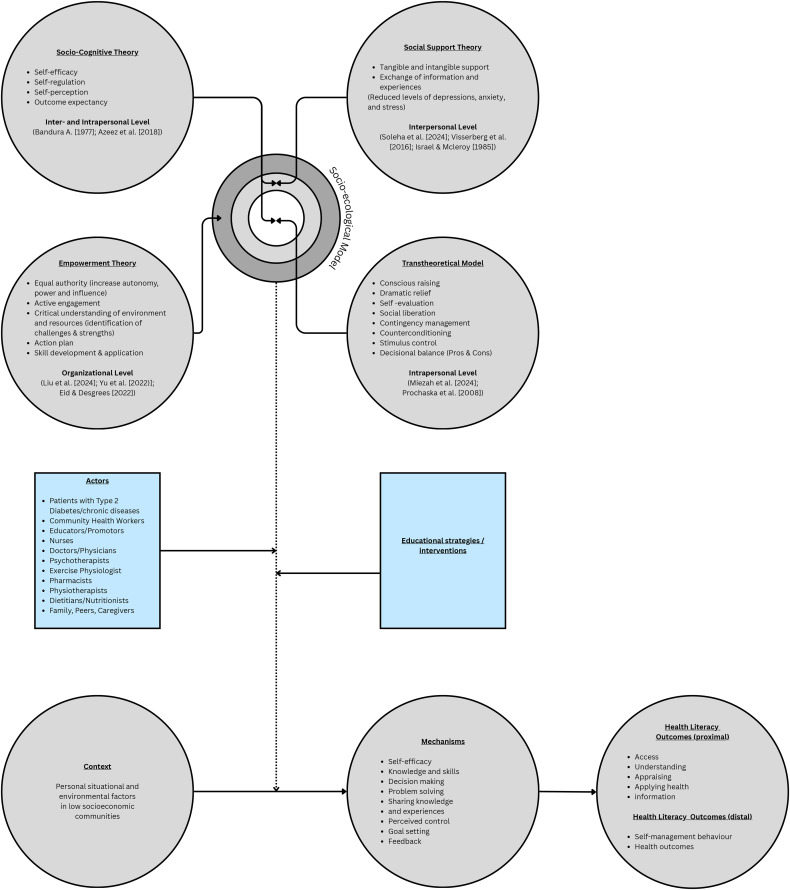
The ICAMO framework.

The proposed ICAMO framework for initial programme theories for educational strategies is presented in Fig 2.

#### Step 3: Search for evidence.

This step aims to search for relevant papers, which will then be further screened and appraised to test and refine the initial programme theories. Therefore, a purposive literature search will be conducted using a revised pretested search strategy informed by the programme theories. The subject headings and keywords will be combined with ‘OR’ and ‘AND’ to ensure relevant literature is retrieved to test and refine the initial programme theories. Search strategies will be formulated collaboratively by the core team and the librarian. An example of a search strategy developed in PubMed is attached (see S2 Table). The following databases will be searched: PubMed, Global Health, Medline, Embase, Education Resource Information Centre (ERIC), PsycINFO, Cumulative Index to Nursing and Allied Health Literature (CINAHL), and Scopus database. Evidence will be drawn from multiple sources to prevent publication bias and ensure that relevant information is collected [69]. Other papers will be identified through:

- Manual searches of reference lists (Citation tracking)
- Manual searches of grey literature, such as the WHO Institutional Repository for information sharing (IRIS), Google Scholar
- Input from core team on other relevant publications, guidelines, or policy papers. Supplementary searches will be by the emerging programme theories, with additional targeted searches conducted as new theoretical questions arise during synthesis.

**Inclusion and exclusion criteria:** The following inclusion criteria will be applied: (i) all study designs on educational strategies to improve health literacy in adults with T2D; (ii) adults (≥ 18 years); (iii) all settings (health care settings and in the community); and (iv) studies done in countries earmarked as low- and middle-income countries by the World Bank for the 2026 fiscal year [70] (v) studies done in low socioeconomic communities or disadvantaged groups in high-income countries*.* The search duration is unrestricted. The inclusion and exclusion criteria may be revised with input from the core team as the review progresses to facilitate the collection of essential components of educational strategies (see Table 3).

**Table 3:** Inclusion and exclusion criteria.

P-Population	People who are 18 years and above, living with type 2 diabetes in low socioeconomic communities
**I**-**Intervention**	Education approaches (Learning strategies)
**P**-**Professionals**	Health providers, including community health workers, peers, family, and caregivers
**O**-**Outcome**	Health literacy: Access, Understanding, Appraisal, and Applying health information and self-management behaviour, criteria, and health outcomes
**H**-**Healthcare setting**	Any healthcare settings and community programmes providing health education to people with low literacy levels in low socioeconomic settings
**Study design**	There are no restrictions on study design. All papers will be included, including grey literature and opinion papers
**Exclusion criteria**	Health education approaches for people receiving palliative care (focuses on symptom relief rather than managing or controlling the condition to prevent complications)
	Papers describing health literacy or self-management that do not employ similar theories to the transtheoretical model, social support, social cognitive, and empowerment theories
	Study protocols
	Studies conducted in high-income countries that do not focus on low socioeconomic communities defined in this study’s inclusion criteria.

#### Step 4: Selection and appraisal of evidence.

Papers will be selected based on their relevance, richness, and rigour to test and refine the programme theories [50,71]. Unlike other traditional studies, where methodological quality, checklists, and methodological hierarchy strictly inform the eligibility criteria [71], the inclusion of papers will mainly be informed by the contribution of the paper to the development, testing, and refinement of the programme theories.

Papers from electronic libraries will be retrieved in text form for appraisal. Duplicates will be removed, and the remaining papers will undergo further screening and evaluation by the core team. Consistency in paper selection will be ensured by applying predefined inclusion and exclusion criteria across all sources. To minimise bias and systematic error, two reviewers, the assistant researcher (MML) and the principal investigator (NMN), will independently select and evaluate 10% of the included and excluded papers by examining the title, abstract, and full text. Reasons for excluding papers will be documented. Disagreements will be resolved through core team consensus, with all decisions documented to ensure transparency [72]. The reviewers will continue checking on each other’s work. Similarly, a reflexive journal will be maintained throughout this review process to document interpretation and critically examine how positionality may have influenced it.

**Relevance:** Relevance in considering papers is defined as the presence of evidence in the papers that addresses the programme theory, contributing to theory development, testing, and refinement [73]. Papers will be considered relevant if they have information on educational strategies and/or mechanisms that influence health literacy in people with T2D or chronic conditions in different contexts and outcomes (refer to the definition of outcomes) [73].

**Richness:** Relevant papers will further be screened for their richness. Richness will be assessed on how much the paper provides information concerning the Intervention-Context- Actor-Mechanism-Outcome configurations (ICAMO) [73]. Papers will be graded as either ‘high’ or ‘low’. ‘High’ papers provide sufficient information on how interventions change contexts, delivered to/by specific actors to trigger a certain response that yields intended or unintended outcomes. Thus, providing sufficient information on how an intervention is working, which includes individuals, social or systems processes, forces, and /or power, and an explanation of the contextual factors that influence it [74]. ‘Low’ papers offer less information on ICAMO. High papers will be included to test rigour further, while the ‘low’ papers will be excluded and kept aside for later consideration [73].

**Fig 3:**
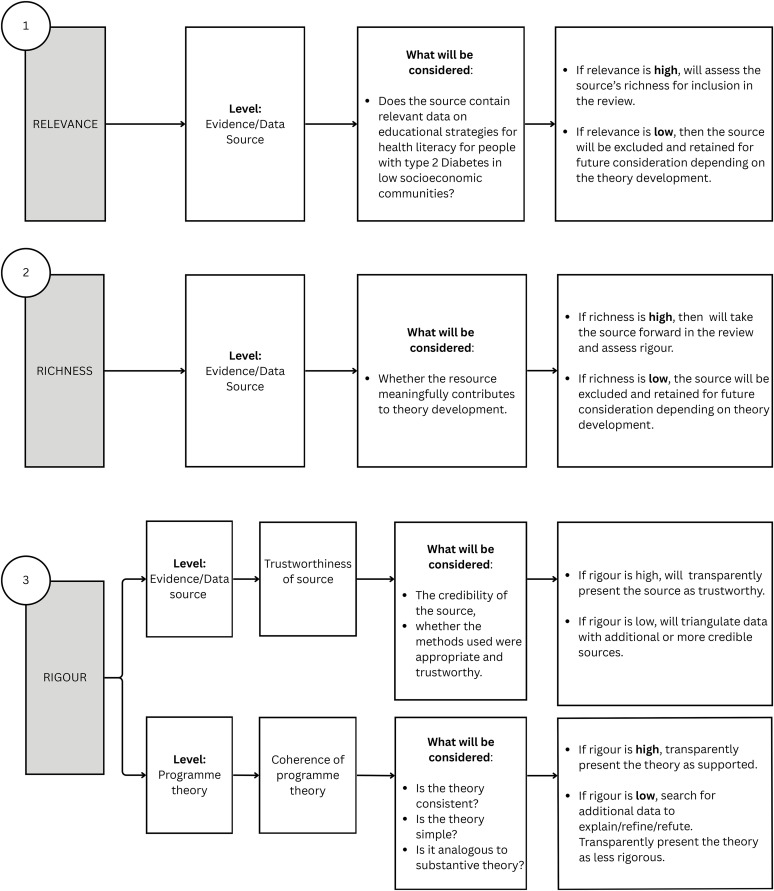
The selection and appraisal of papers for relevance, richness, and rigour.

**Rigour:** Rigour will be tested at the programme theory and data source level [40,71]. At the programme theory level, only studies with similar theories to the theories under test will be selected [34]. For the data source level, we will consider the credibility of the sources, including the appropriateness and trustworthiness of the methods used. It will involve checking the credibility of the conclusion made by the original authors and the methodology of the study, including participant selection, methods of data collection, sample size, and data analysis [40,50]. However, papers with low rigour will also be considered if they are relevant and rich enough to refine the programme theory [71]. Evidence from low-rigour sources that have relevant information will be triangulated with other sources with rigour if the information is consistent. The core team will assess the quality of the papers. Fig 3, adapted from Dada et al. [73], illustrates the procedure that will be undertaken to select and appraise papers for the review: -

#### Step 5: Data extraction and synthesis.

At this stage, the core team will lead the process of data extraction, analysis, and synthesis. The support review team will contribute only at the final stage by reviewing the draft of the final Initial programme theories and the narrative development of middle-range theories (see Fig 1).

**Data extraction:** Data extraction will be conducted independently by the principal and assistant researcher, with each reviewing the other’s entries for accuracy and consistency. Regular meetings with the core team (SH, MH, MM, FG) will be held to discuss progress and any issues with data extraction [40].

A Microsoft Excel sheet embedded with programme theories will be developed to facilitate data collection. The sheet will be piloted on each type of evidence source before utilisation to ensure consistency and efficiency in data extraction. This extraction sheet will be populated with the Intervention-Context-Actors-Mechanism-and Outcomes from various articles. It will capture study characteristics (Title, Author, Publication year, country, study setting, participants’ characteristics, sample size, study objectives, and design) as well as findings. Study findings, as earlier stated, will include themes such as educational strategies (**Interventions**), barriers and facilitators of health literacy (**Context)**, **Actors,** human and social systems processes, forces, powers (**Mechanisms**), and **Outcomes**. Therefore, any ICAMO components in the included studies will be extracted and entered into the data sheet using both inductive and deductive approaches.

Data to test and refine the programme theories will be extracted from selected papers. However, the search for papers will continue until theories are refuted or supported with sufficient (relevant, rich, and rigorous) evidence [75]. The data extraction sheet may be revised to include any additional information identified as important during the process (see Table 4).

**Table 4:** Proposed data extraction sheet with embedded guidance.

Information of Interest	Definition
**Author**	, Davies & Kenny or Davies et. al. (for multiple authors)
**Year**	The year the article was published
**Publication**	Where the article was published (e.g., Health Literacy Research and Practice)
	For organisation guidelines, e.g., the World Health Organisation website, American Diabetes Association (ADA) – Standards of Care in Diabetes
**Country**	The Country where the study was conducted
**Date data were collected**	The date or period when the data were collected. If no date or period is indicated, then write N/A
**Aim of the study**	The main objective(s) of the study
**Type of evidence source**	Primary research: peer-reviewed research articles, randomised controlled trials
	Discussion articles
	Epidemiology papers
	Evidence synthesis, systematic reviews, realist reviews, scoping reviews, etc.
	Conference abstracts
	Editorials
	Thesis
	Guidelines
**Design**	Qualitative/Quantitative/Mixed
**Sample size**	Number of participants
**Participants’ characteristics**	If stated: Characteristics of those included in the study, e.g., the condition (disease), presence of comorbidity, newly diagnosed/old cases
**Age (yrs)**	The age/age group (range)
**Sex**	F/M, not disclosed
**Other demographics**	Socioeconomic status
**Study Setting**	Rural, Urban, or Peri-urban
**Educational strategies**	Type/Content of the intervention
**Timing of Delivery of Programme**	At diagnosis
	During routine care delivery (review days)
	When not meeting set goals
	When new complications set in
	Yearly etc.
**Place of delivery**	In the community or at the health facility
**The individual delivering the intervention**	Trained Health Providers (Nurses, Pharmacists, etc)
	Community health workers (trained/experienced)
	Caregivers (family/volunteers)
**Mode of delivery (Format)**	In person, Virtual, Group or Individual (one-on-one)
**Medium of delivery (tools/channels used)**	Physical
	Illustrated materials with texts
	Printed materials or Visual aids
	Telecommunication information technologies and devices (videos, SMS, YouTube, Facebook, WhatsApp)
**Methods of delivery (teaching methods)**	Role play, Discussion, Teach back, Lectures, Demonstrations, Counselling/Coaching, etc.
**Duration of Intervention**	Length of time/day/session
	Duration of programme (if applicable)
**Underpinning theory (if present)**	Transtheoretical, Social support, social cognitive, and empowerment theory, Others or Not applicable
**Mechanisms (Processes/responses to educational strategy)**	Knowledge, skills, self-efficacy, confidence, feedback, motivation, decision-making
**Outcomes (Positive/Negative)**	Knowledge, Self-management behaviour,
	Clinical outcomes
	Understanding one’s health, health information, and the health care system
	Appraise and utilise health information
	Health-seeking behaviour
	Collaborating with health providers
	Self-care activities
	Biomedical, fasting glucose level, body weight, BMI, etc
**Evidence of configured ICAMO elements**	E.g. ICAMO 1, ICAMO2, ICAMO 3 etc
	E.g. I1-C1-A1-O1; I1-C2-A2-M2-O2; I2-C3-A2-M3-O3 etc
	E.g., I-C, I-O
	E.g. I C-A M

**Data analysis and synthesis:** The process of analysis and synthesis will be iterative, as the reviewers will collate the emerging themes to form chains of inference across papers. Data will be analysed through realist logic analysis using the ICAMO configuration. It will be tested and refined by examining various interventions, contexts, actors, and mechanisms, identified from different sources, to identify patterns of causality [76,77]. The analytic process will follow the realist evaluation approach, as in Rycroft-Malone [78] and is outlined below:

a Extracted data will be merged into evidence tables
b Theming by individual reviewers for each article
c Reviewers’ themes for each specific article will be compared and used to formulate chains of inference
d Eventually, refined programme theory propositions will be formulated

A chain of inference is defined as ‘a connection that can be made across articles based on themes identified’ [78]. This means that each theme extracted from an article is coded as relating to I, C, A, M, or O, and chains of inference are built by connecting and triangulating themes across articles that together constitute an empirically supported and theoretically plausible causal relationship [78]. Unclear and conflicting themes will be addressed through a detailed re-examination of relevant papers with the core team to seek an explanation. Where applicable, methods such as juxtaposing, reconciling, situating, and adjudicating will be employed to resolve unclear themes and explanations [75]. Retroductive reasoning will be employed to uncover causal forces that lie behind identified patterns, explaining how educational strategies may change the context in ways that trigger the underlying mechanisms to produce intended or unintended health literacy outcomes [76,78]. The findings will be reviewed, and a draft of the final programme theories will be presented virtually to the support review team for feedback.

**Develop a narrative and disseminate:** At this stage, the entire realist review team will have virtual meetings to discuss and compile middle-range theories from the draft of the final programme theories. Finally, a narration of middle-range theories that are transferable to different settings and evidence-based will be compiled to inform practice, programmes, and policy for improving the health literacy of people with T2D in low socioeconomic communities.

#### Ethical statement and declarations.

No ethical clearance is required as this review will primarily utilise secondary data. Nevertheless, all reviewers participating in this study are qualified and experienced in their respective roles. They have stated that no competing interests exist.

#### The Status and time of the study.

This review remains at the protocol stage, with the first two steps (Steps 1 and 2) already completed

## Discussion

To our knowledge, this will be the first realist review to examine and provide an understanding of educational strategies for improving health literacy in individuals with type 2 diabetes in low socioeconomic communities. The findings could be transferable to other chronic conditions in similar settings. This transferability of findings will be possible due to realist reviews offering explanations as to why and how interventions work in specific contexts and not others that emanate from theoretical thinking and empirical evidence [79].

The study may also bridge the gap between research and the implementation of evidence. This gap often arises from challenges in translating research findings into real-world settings and determining whether programmes (including certain education strategies) may work in some contexts. Realist review helps to address this challenge by providing the necessary explanations of what works or does not work, in what circumstances, for whom, how, and why, to ensure that strategies are implemented to the right people, in the right place, using appropriate methods, and at the right time [80].

The study may provide clear, evidence-based guidance on how to implement health literacy programmes for people with T2D and their caregivers across diverse settings. It aims to empower individuals in vulnerable communities with the knowledge and skills needed to manage their condition and prevent complications. It may also identify which strategies are less effective for those with lower education levels and suggest ways to improve understanding and use of health information. The findings could support health providers and policymakers in designing inclusive educational strategies that enable people from low socioeconomic backgrounds to access, understand, and apply health information effectively. It may also encourage active participation in care decisions, contributing to the Sustainable Development Goal of ‘leaving no one behind’, thus reducing health inequities [81].

### Anticipated challenges

The researchers may encounter challenges in developing programme theories, testing, and refining them to create transferable strategies. This will be minimised through in-depth discussions among the review team and the iterative nature of the process, supported by the multi-disciplinary collaboration of team members with relevant expertise. Another anticipated challenge is the significant time required to conduct an iterative type of research, in which the core team will be engaged more regularly to minimise this challenge. Finally, the fact that only a few studies on health literacy interventions have clear theoretical frameworks may pose a challenge [82]. However, further data searches will be conducted on these papers to identify similarities with those presenting clear theories. Other sources of information will also be searched to test and refine the Initial Programme Theory.

### Limitations of the study design

People from the lowest socioeconomic groups are often underrepresented in existing literature, making it challenging to determine their experiences, preferences, or to identify the most effective strategies to enhance their health literacy.

### Dissemination plans

Study results, including the ICAMO coding framework, will be made public. Dissemination of results will be through seminars and conferences. The results will be similarly published in a peer-reviewed journal to guide practice, programme development, and policy in related areas. These findings will also inform the co-creation of a Diabetes Self-Management Education and Support programme tailored to low socioeconomic communities in Zambia, to be integrated into the PEN-Plus model of care.

### Amendments to the study

Any amendments that may be required during the study process for the successful completion of the review will be presented to all authors and agreed upon before implementing the changes. Modifications will be documented through an audit trail and reported in the findings, including the rationale for these changes.

## Supporting information

S1 Table RAMESES publication standards: realist synthesis. (PDF)

S2 Table PubMed search Query. (DOCX)
