# Supplementary material for: Educational strategies to improve health literacy for people with type 2 diabetes in low socio-economic communities: A realist review protocol
Source: PLoS One. 2026 Jul 30;21(7):e0352599. doi: 10.1371/journal.pone.0352599 (PMC13422839; doi:10.1371/journal.pone.0352599)
Supplement: S2 Table — (DOCX) [file pone.0352599.s002.docx]

**S 2: PubMed Search Strings (17/05/2026)**

| **No.** | **Theory** | **Query String** | **Results** |
| --- | --- | --- | --- |
| **#1** | **Intrapersonal Level**  Transtheoretical and Patient Activation Theory | ("Transtheoretical Model"[All Fields] OR "Patient Activation"[All Fields] OR "person centred"[All Fields] OR (("individual s"[All Fields] OR "individualisation"[All Fields] OR "individualise"[All Fields] OR "individuations"[All Fields] OR "persons"[MeSH Terms] OR "persons"[All Fields] OR "individual"[All Fields]) AND ("stage"[All Fields] OR "staged"[All Fields] OR "stages"[All Fields] OR "staging"[All [All Fields] OR "pattern"[All Fields] OR "pattern s"[All Fields] OR "patternability"[All Fields] OR "patternable"[All Fields] OR "patterned"[All Fields] OR "patterning"[All Fields] OR "patternings"[All Fields] OR "patterns"[All Fields]) AND ("change"[All Fields] OR "changed"[All Fields] OR "changes"[All Fields] OR "changing"[All Fields] OR "changings"[All Fields])) OR ("self efficacy"[MeSH Terms] OR ("self"[All Fields] AND "efficacy"[All Fields]) OR "self efficacy"[All Fields]) OR "self-management behaviour"[All Fields] OR "decision making"[All Fields] OR "Changes stages"[All Fields] OR ("Transtheoretical Model"[MeSH Terms] OR ("transtheoretical"[All Fields] AND "model"[All Fields]) OR "Transtheoretical Model"[All Fields] OR ("model"[All Fields] AND "transtheoretical"[All Fields]) OR "model transtheoretical"[All Fields]) OR ("self efficacy"[MeSH Terms] OR ("self"[All Fields] AND "efficacy"[All Fields]) OR "self efficacy"[All Fields] OR ("efficacy"[All Fields] AND "self"[All Fields]) OR "efficacy self"[All Fields])) AND "Self concept"[All Fields]) OR "Behaviour and Behaviour Mechanisms"[All Fields] OR "Personality development"[All Fields] OR "Patient Centered Care"[All Fields] OR "Situational Awareness"[All Fields] OR "Patient Involvement"[All Fields] OR "Self Management"[All Fields] OR "Self-Management Programs"[All Fields] OR "Self-care"[All Fields] OR "self-medication"[All Fields] OR ("Self Management"[MeSH Terms] OR "Self Management"[All Fields] OR ("program"[All Fields] AND "self"[All Fields] AND "management"[All Fields]) OR "program self management"[All Fields])) AND "Diabetes Mellitus"[All Fields]) OR "Adult-Onset"[All Fields]) AND "diabetes mellitus type ii"[All Fields]) OR "Low Socioeconomic Level"[All Fields] OR "communication health"[All Fields] OR ("health literacy"[MeSH Terms] OR ("health"[All Fields] AND "literacy"[All Fields]) OR "health literacy"[All Fields] OR ("literacy"[All Fields] AND "health"[All Fields]) OR "literacy health"[All Fields]) OR OR ("sensitive"[All Fields] AND "population"[All Fields] AND "groups"[All Fields]) OR "sensitive population groups"[All Fields])) AND ("low"[All Fields] AND ("developing countries"[MeSH Terms] OR ("developing"[All Fields] AND "countries"[All Fields]) OR "developing countries"[All Fields] OR ("middle"[All Fields] AND "income"[All Fields] AND "countries"[All Fields]) OR "middle income countries"[All Fields]))) OR "Developing Country"[All Fields]) AND "Adults"[All Fields] | 803 |
| **#2** | **Interpersonal Level**  Social cognitive theory | ("Social cognitive theory"[All Fields] OR ("self efficacy"[MeSH Terms] OR ("self"[All Fields] AND "efficacy"[All Fields]) OR "self efficacy"[All Fields]) OR ("motivate"[All Fields] OR "motivated"[All Fields] OR "motivates"[All Fields] OR "motivating"[All Fields] OR "motivation"[MeSH Terms] OR "motivation"[All Fields] OR "motivations"[All Fields] OR "motive"[All Fields] OR "motivational"[All Fields] OR "motivator"[All Fields] OR "motivators"[All Fields] OR "motives"[All Fields]) OR ("confidences"[All Fields] OR "confident"[All Fields] OR "confidently"[All Fields] OR "confidents"[All Fields] OR "self concept"[MeSH Terms] OR ("self"[All Fields] AND "concept"[All Fields]) OR "self concept"[All Fields] OR "confidence"[All Fields]) OR "role models"[All Fields] OR "Psychological Theories"[All Fields] OR ("psychological theory"[MeSH Terms] OR ("psychological"[All Fields] AND "theory"[All Fields]) OR "psychological theory"[All Fields] OR ("theories"[All Fields] AND "social"[All Fields] AND "cognitive"[All Fields]) OR "theories social cognitive"[All Fields]) OR "Health Communications"[All Fields] OR "communication health"[All Fields] OR "Diabetes Mellitus"[All Fields]) AND ()) OR "Adult-Onset"[All Fields] OR "diabetes mellitus adult onset"[All Fields] OR "Diabetes Mellitus"[All Fields]) AND ()) OR "Adult-Onset"[All Fields]) AND "Low Socioeconomic Level"[All Fields]) OR "literacy health"[All Fields] OR "Health Communications"[All Fields] OR "communication health"[All Fields]) AND "Disadvantaged Populations"[All Fields]) OR "Sensitive Population Groups"[All Fields]) AND ("low"[All Fields] AND ("developing countries"[MeSH Terms] OR ("developing"[All Fields] AND "countries"[All Fields]) OR "developing countries"[All Fields] OR ("middle"[All Fields] AND "income"[All Fields] AND "countries"[All Fields]) OR "middle income countries"[All Fields]))) OR ("developing countries"[MeSH Terms] OR ("developing"[All Fields] AND "countries"[All Fields]) OR "developing countries"[All Fields] OR ("country"[All Fields] AND "developing"[All Fields]) OR "country developing"[All Fields]) OR "Developing Country"[All Fields]) AND "Adults"[All Fields] | 7,998 |
| **#3** | **Interpersonal level**  Social support theory | ("Social support"[All Fields] OR "Environmental change"[All Fields] OR "emotional support"[All Fields] OR "psychological support"[All Fields] OR "physical support"[All Fields] OR "support social"[All Fields] OR "Perceived Social Supports"[All Fields] OR "support online social"[All Fields] OR ("Social support"[MeSH Terms] OR ("social"[All Fields] AND "support"[All Fields]) OR "Social support"[All Fields] OR ("care"[All Fields] AND "social"[All Fields]) OR "care social"[All Fields]) OR "Family Supports"[All Fields] OR "encouragement family"[All Fields] OR "environments social"[All Fields] OR "contexts social"[All Fields]) AND "Health Communications"[All Fields]) OR "communication health"[All Fields]) AND "diabetes mellitus type ii"[All Fields]) OR "Diabetes Mellitus"[All Fields]) AND ()) OR "Adult-Onset"[All Fields]) AND "Low Socioeconomic Level"[All Fields]) OR "literacy health"[All Fields] OR "Disadvantaged Populations"[All Fields] OR "Sensitive Population Groups"[All Fields]) AND ("low"[All Fields] AND ("developing countries"[MeSH Terms] OR ] OR ("country"[All Fields] AND "developing"[All Fields]) OR "country developing"[All Fields]) OR "Developing Country"[All Fields]) AND "Adults"[All Fields] | 8006 |
| **#4** | **Organisational Level**  Empowerment theory | ("Empowerment theory"[All Fields] OR "critical awareness"[All Fields] OR "Active participation"[All Fields] OR "decision making"[All Fields] OR "self-management"[All Fields] OR "equal authority"[All Fields] OR "environmental change"[All Fields] OR "self-determination"[All Fields] OR "Free will"[All Fields] OR ("personal autonomy"[MeSH Terms] OR ("personal"[All Fields] AND "autonomy"[All Fields]) OR "personal autonomy"[All Fields] OR ("autonomy"[All Fields] AND "personal"[All Fields]) OR "autonomy personal"[All Fields]) OR "Diabetes Mellitus"[All Fields]) AND ()) OR "Adult-Onset"[All Fields]) AND "diabetes mellitus type ii"[All Fields]) OR "Low Socioeconomic Level"[All Fields]) AND "Health Communications"[All Fields]) OR "communication health"[All Fields] OR "literacy health"[All Fields] OR "Disadvantaged Populations"[All Fields] OR "Sensitive Population Groups"[All Fields]) AND ("low"[All Fields] AND ("developing countries"[MeSH Terms] OR OR "country developing"[All Fields]) OR "Developing Country"[All Fields]) AND "Adults"[All Fields] | 8,007 |
| **#5** | All theories | Search: **#1 AND #2 AND #3 AND #4** | 626 |
