# Supplementary material for: Educational strategies to improve health literacy for people with type 2 diabetes in low socio-economic communities: A realist review protocol
Source: PLoS One. 2026 Jul 30;21(7):e0352599. doi: 10.1371/journal.pone.0352599 (PMC13422839; doi:10.1371/journal.pone.0352599)
Supplement: Attachment — Submitted filename: 1. PLOS ONE Reviewer Repor.docx..docx [file pone.0352599.s003.docx]

**PLOS ONE Reviewer Report**

**Study protocol: Educational strategies to improve health literacy for people with type 2 diabetes in low socio-economic communities: A realist review protocol**

**Reviewer Comments to the Author**

**1. General Assessment**

This manuscript addresses an important and timely topic with clear relevance to public health, health equity, and chronic disease management. Improving health literacy among people with type 2 diabetes in low socio-economic communities is highly aligned with global priorities for noncommunicable disease (NCD) control and reducing health inequities.

The manuscript presents a coherent rationale for conducting a realist review and identifies a meaningful gap in understanding how and why educational strategies work in disadvantaged contexts. The focus on mechanisms and contextual influences is appropriate for this field.

However, several areas require clarification and strengthening, particularly regarding realist methodology, conceptual framing, and alignment with global health promotion frameworks.

**2. Major Comments**

**2.1 Conceptual Framing and Theoretical Foundation**

While the problem statement is clearly articulated, the manuscript would benefit from stronger conceptual grounding. Specifically:

- The initial programme theory should be more explicitly articulated.
- The authors should clarify the theoretical assumptions underpinning how educational strategies are expected to influence health literacy and diabetes outcomes.
- Greater clarity is needed regarding how Context–Mechanism–Outcome (CMO) configurations will be developed and refined.

From a global health promotion perspective, the study could be strengthened by referencing principles articulated in the Ottawa Charter for Health Promotion, particularly:

- Developing personal skills
- Creating supportive environments
- Strengthening community action
- Building healthy public policy
- Reorienting health services

Educational strategies for diabetes management in low socio-economic communities inherently intersect with these action areas. Explicitly situating the realist inquiry within this broader health promotion framework would enhance theoretical coherence and policy relevance.

**2.2 Realist Methodology and Rigor**

As this is a realist review protocol, the manuscript should clearly demonstrate adherence to established realist standards, such as the RAMESES publication guidelines.

The authors should clarify:

- How the realist approach is justified over alternative review methods.
- How the initial programme theory was developed.
- How CMO configurations will be identified, tested, and refined.
- Whether stakeholder engagement will be used to refine programme theory.
- How iterative searching will be conducted.
- How relevance and rigour of included studies will be appraised (rather than relying solely on traditional hierarchies of evidence).

Greater specificity in these areas will improve transparency and methodological credibility.

**2.3 Consideration of Social and Commercial Determinants of Health**

Given the focus on low socio-economic communities, the review should more explicitly engage with:

- Social determinants of health (income, education, employment, housing stability)
- Structural barriers to diabetes self-management
- Health system accessibility constraints
- Potential commercial determinants influencing diabetes-related behaviours

Health literacy interventions do not operate in isolation; they are embedded within structural and systemic contexts. Integrating a systems lens would strengthen the explanatory power of the realist review.

**2.4 Equity Considerations**

Equity is central to this topic. The manuscript should clarify:

- How equity-sensitive outcomes will be identified.
- Whether differential effectiveness across subgroups will be explored.
- How contextual disadvantage will be conceptualised within CMO configurations.
- Whether the review will explore risk of intervention-generated inequity.

A stronger articulation of equity analysis would enhance the contribution of the study.

**2.5 Methodological Clarity and Replicability**

Although the methods are described in reasonable detail, further clarification is needed to ensure replicability:

- Provide detailed inclusion and exclusion criteria.
- Specify databases and full search strategy.
- Clarify screening procedures and inter-rater processes.
- Describe data extraction frameworks.
- Clarify how disagreements will be resolved.
- Specify analytic software (if applicable).
- Describe reflexivity considerations in interpretation.

Given that realist reviews are iterative, the authors should explain how deviations from the initial protocol will be documented.

**2.6 Data Management and Transparency**

The manuscript should provide a clearer Data Availability statement consistent with PLOS policy. Specifically:

- Indicate where extracted data and analytic materials will be deposited.
- Clarify whether coding frameworks and CMO configurations will be publicly shared.
- Describe how transparency will be ensured throughout theory development.

**3. Minor Comments**

- Some sections would benefit from tighter language and reduced repetition.
- Define key terms (e.g., health literacy, mechanism, context) at first use.
- Ensure consistency in terminology across sections.
- Consider including more recent global references (2022–2024).
- Ensure alignment between stated objectives and anticipated outputs.

**4. Relevance and Contribution**

This protocol addresses a highly relevant topic at the intersection of diabetes management, health literacy, and social inequity. A realist approach is appropriate given the complexity of educational interventions in disadvantaged settings.

With strengthened methodological detail, clearer articulation of programme theory, and stronger integration of health promotion and equity frameworks, this review has strong potential to contribute meaningfully to both academic literature and policy discussions.
