# Supplementary material for: Educational strategies to improve health literacy for people with type 2 diabetes in low socio-economic communities: A realist review protocol
Source: PLoS One. 2026 Jul 30;21(7):e0352599. doi: 10.1371/journal.pone.0352599 (PMC13422839; doi:10.1371/journal.pone.0352599)
Supplement: Attachment — Submitted filename: Responses to Reviewers comments.docx [file pone.0352599.s005.docx]

| **PLOS ONE REVIEWERS’ REPORT**  **Study protocol: Educational strategies to improve health literacy for people with type 2 diabetes in low socio-economic communities: A realist review protocol** | | |
| --- | --- | --- |
| **REVIEWERS’ COMMENTS** | **AUTHORS’ RESPONSE** | **PAGE No. AND LINES** |
| **REVIEWER 1** |  |  |
| 2.1 **Conceptual Framing and Theoretical Foundation**   - The initial programme theory should be more explicitly articulated. - The authors should clarify the theoretical assumptions underpinning how educational strategies are expected to influence health literacy and diabetes outcomes. - Greater clarity is needed regarding how Context–Mechanism–Outcome (CMO) configurations will be developed and refined.   From a global health promotion perspective, the study could be strengthened by referencing principles articulated in the Ottawa Charter for Health Promotion, particularly:   - Developing personal skills | The conceptual framework, which is the initial programme theory, has been added to the document **(see Fig. 3).** The process of the development of the framework has also been included in **steps 1 and 2.**  The theoretical assumption underpinning the educational strategies emanating from programme theories has been stated (**see also Table 2)**  ICAMO has been clearly explained with **Table 1** attached for more information  Steps 1 and 2 show how ICAMO was developed, that is, the initial programme theories and the refining is explained under data analysis  The study aims to improve self-management behaviour by strengthening knowledge and skills, which are within the domain of personal skills development. The recent paper for WHO on health literacy development was referenced. | Pg. 7 (225) – 9 (263)  Page 11, lines 323 - 330  Page 6, lines 189 - 204  Pg. 8 (225) – 9 (263)  Page 16, Lines 488 - 516 |
| **2.2 Realist Methodology and Rigor**  - How a realist review is justified  - How the initial programme theory was developed  How CMO will be identified, tested  - Whether stakeholders will be used  - How the relevance and rigour of appraised studies will be appraised  The authors should clarify how this work builds upon, diverges from, or fills limitations in prior studies. A more explicit statement of the expected contribution—whether methodological, theoretical, empirical, or policy-oriented—would further strengthen the justification for the study | The justification of realist review has been elaborated in the introduction.  Development of the programme theory is explained under steps 1 and 2  Explanation of how CMO will be developed and tested is provided  The study will utilise secondary data, and it will be conducted by the review team, who are experts in various fields.  No primary data will be obtained from stakeholders  The detailed explanation of how relevance and rigour will be appraised is provided **(see also Fig. 4)**  Clarification was provided.  The study is **employing a realist review** approach, with particular attention to people living in low-socioeconomic communities | Page 4, lines 125 - 137  Pages 8 – 9, lines 225 – 263  Page 16, Lines 488 - 516  Page 6, lines 167 – 180  Page 13, Lines 404 - 452  Pg. 4, Lines 125 - 133 |
| 2.4 **Equity Consideration** | The study incorporates equity considerations by focusing on low‑socioeconomic communities. It aims to examine barriers to effective self‑management (context) and to explore strategies across different settings to overcome these barriers, thereby promoting better health outcomes for all | Pg. 5, Line 130 -133 |
| 2.5 **Methodological Clarity and Replicability**  Provide detailed inclusion and exclusion criteria  Describe the reflexivity consideration in interpretation  Given that realist reviews are iterative, the authors should explain how deviations from the initial protocol will be documented | Detailed inclusion and exclusion criteria have been provided (**see also Table 3)**  The description has been strengthened.  Documentation of our reflections and decision-making process will be maintained throughout this review to account for Reflexivity  The authors explained under **Amendments to the study** how deviation from the protocol will be addressed. | Pg. 13, Lines 393 - 400  Page 14, Lines 418 -420  Page 19, lines 581 - 584 |
| **2.6 Data Management and Transparency**   - Clarify whether coding frameworks and CMO configurations will be publicly shared. - Describe how transparency will be ensured throughout theory development | All the results will be shared  It has been added under Dissemination plans  The inclusion and exclusion criteria and the programme theories will guide the process of selecting papers, data extraction, and analysis. The reviewers will consistently check each other's work during the process. Any differences will be resolved by the core team, and the rationale behind any decision made will be documented. | Page: 19, lines 574 - 579  Page 13. lines 412 – 320  Page 15. 460 – 463  Pg. 16. Lines 510 - 511 |
| **2.3 Consideration of social and commercial determinants of health** | The third paragraph discusses contextual factors, which are the social determinants of health that contribute to health inequities.  To emphasize this point, we have added the terms social determinant of health and health inequality  The development of the initial programme theory (the conceptual framework) has also included a SDOH lens | Page. 3 lines 85 – 97  Pg 3, line 87.  Pg 11. lines 323 - 330 |
| **(clarify whether screening and extraction will be conducted independently by multiple reviewers and how disagreements will be resolved to ensure transparency)** | Data extraction will be conducted independently by the principal investigator and the assistant researcher, with each reviewing the other’s entries for accuracy and consistency. Regular meetings with the core team will be held to discuss progress and any issues with data extraction and analysis. | Pg 13. Lines 413 - 420  Page 15, lines 460 - 463 |
| Comments from reviewer 2 |  |  |
| The search strategy is described broadly; the manuscript should provide a more reproducible search approach, including clearer operationalisation of search terms, database strategy, and iterative search procedures | The search terms will be systematically derived from the study hypotheses to ensure alignment with the review objectives. In collaboration with the librarian (AM), these terms will be carefully developed and pilot-tested before their formal implementation in **Step 3** of the review process. We have attached an example of the PubMed pretested search string | See Supplementary file, S 2  Page 12, lines 369 - 374 |
| The processes for study selection, appraisal, and data extraction need sharper detail, particularly how relevance, richness, and rigour will be judged consistently across reviewers. | To ensure consistency across reviewers, predefined inclusion/exclusion criteria will be applied. The programme theories will similarly guide the appraisal process. No checklists will be utilised to prevent the exclusion of relevant papers. The reviewers will consistently check each other's work.  All decisions to exclude papers will be transparently documented to strengthen credibility (**see also Fig 4 & Data extraction sheet Table 4)** | Page 13, lines 412 - 420  Page 15, lines 460 - 477 |
| The analytic section would also be strengthened by clarifying how ICAMO configurations will be developed from extracted data and how competing explanations will be handled. | The process of how ICAMO will be developed from the extracted data has been outlined.  Competing explanations will be systematically revisited by the core team through re-examination of relevant papers. | Pg. 16, lines 489 - 511 |
