# Supplementary material for: Educational strategies to improve health literacy for people with type 2 diabetes in low socio-economic communities: A realist review protocol
Source: PLoS One. 2026 Jul 30;21(7):e0352599. doi: 10.1371/journal.pone.0352599 (PMC13422839; doi:10.1371/journal.pone.0352599)
Supplement: Peer review history

# Peer review: Decision Letter 0

**DOI:** 10.1371/journal.pone.0352599.r001

**Authors:** Marwan Al-Nimer

4 Apr 2026

PONE-D-25-49650

Educational strategies to improve health literacy for people with type 2 diabetes in low socio-economic communities: A realist review protocol

PLOS One

Dear Dr.  Heine,

Thank you for submitting your manuscript to PLOS ONE. After careful consideration, we feel that it has merit but does not fully meet PLOS ONE’s publication criteria as it currently stands. Therefore, we invite you to submit a revised version of the manuscript that addresses the points raised during the review process.

Please submit your revised manuscript by May 19 2026 11:59PM. If you will need more time than this to complete your revisions, please reply to this message or contact the journal office at plosone@plos.org. When you're ready to submit your revision, log on to https://www.editorialmanager.com/pone/ and select the 'Submissions Needing Revision' folder to locate your manuscript file.

Please include the following items when submitting your revised manuscript:

- A letter that responds to each point raised by the academic editor and reviewer(s). You should upload this letter as a separate file labeled 'Response to Reviewers'.
- A marked-up copy of your manuscript that highlights changes made to the original version. You should upload this as a separate file labeled 'Revised Manuscript with Track Changes'.
- An unmarked version of your revised paper without tracked changes. You should upload this as a separate file labeled 'Manuscript'.

If you would like to make changes to your financial disclosure, please include your updated statement in your cover letter. Guidelines for resubmitting your figure files are available below the reviewer comments at the end of this letter.

If applicable, we recommend that you deposit your laboratory protocols in protocols.io to enhance the reproducibility of your results. Protocols.io assigns your protocol its own identifier (DOI) so that it can be cited independently in the future. For instructions see: https://journals.plos.org/plosone/s/submission-guidelines#loc-laboratory-protocols. Additionally, PLOS ONE offers an option for publishing peer-reviewed Lab Protocol articles, which describe protocols hosted on protocols.io. Read more information on sharing protocols at https://plos.org/protocols?utm_medium=editorial-email&utm_source=authorletters&utm_campaign=protocols.

We look forward to receiving your revised manuscript.

Kind regards,

Marwan Salih Al-Nimer, MD, PhD

Academic Editor

PLOS One

Journal Requirements:

When submitting your revision, we need you to address these additional requirements.

1.Please ensure that your manuscript meets PLOS ONE's style requirements, including those for file naming. The PLOS ONE style templates can be found at

https://journals.plos.org/plosone/s/file?id=wjVg/PLOSOne_formatting_sample_main_body.pdf and

https://journals.plos.org/plosone/s/file?id=ba62/PLOSOne_formatting_sample_title_authors_affiliations.pdf

2. Please provide a complete Data Availability Statement in the submission form, ensuring you include all necessary access information or a reason for why you are unable to make your data freely accessible. If your research concerns only data provided within your submission, please write "All data are in the manuscript and/or supporting information files" as your Data Availability Statement.

3. Please ensure that you refer to Figure 2 in your text as, if accepted, production will need this reference to link the reader to the figure.

4. If the reviewer comments include a recommendation to cite specific previously published works, please review and evaluate these publications to determine whether they are relevant and should be cited. There is no requirement to cite these works unless the editor has indicated otherwise.

Additional Editor Comments:

**Major Revision**

[Note: HTML markup is below. Please do not edit.]

Reviewer's Responses to Questions

**Comments to the Author**

1. Does the manuscript provide a valid rationale for the proposed study, with clearly identified and justified research questions?

The research question outlined is expected to address a valid academic problem or topic and contribute to the base of knowledge in the field.

Reviewer #1: Yes

Reviewer #2: Yes

**********

2. Is the protocol technically sound and planned in a manner that will lead to a meaningful outcome and allow testing the stated hypotheses?

The manuscript should describe the methods in sufficient detail to prevent undisclosed flexibility in the experimental procedure or analysis pipeline, including sufficient outcome-neutral conditions (e.g. necessary controls, absence of floor or ceiling effects) to test the proposed hypotheses and a statistical power analysis where applicable. As there may be aspects of the methodology and analysis which can only be refined once the work is undertaken, authors should outline potential assumptions and explicitly describe what aspects of the proposed analyses, if any, are exploratory.

Reviewer #1: Partly

Reviewer #2: Partly

**********

3. Is the methodology feasible and described in sufficient detail to allow the work to be replicable?

Descriptions of methods and materials in the protocol should be reported in sufficient detail for another researcher to reproduce all experiments and analyses. The protocol should describe the appropriate controls, sample size calculations, and replication needed to ensure that the data are robust and reproducible.

Reviewer #1: Yes

Reviewer #2: Yes

**********

4. Have the authors described where all data underlying the findings will be made available when the study is complete?

The PLOS Data policy requires authors to make all data underlying the findings described in their manuscript fully available without restriction, with rare exception, at the time of publication. The data should be provided as part of the manuscript or its supporting information, or deposited to a public repository. For example, in addition to summary statistics, the data points behind means, medians and variance measures should be available. If there are restrictions on publicly sharing data—e.g. participant privacy or use of data from a third party—those must be specified.

Reviewer #1: Yes

Reviewer #2: Yes

**********

5. Is the manuscript presented in an intelligible fashion and written in standard English?

PLOS ONE does not copyedit accepted manuscripts, so the language in submitted articles must be clear, correct, and unambiguous. Any typographical or grammatical errors should be corrected at revision, so please note any specific errors here.

Reviewer #1: Yes

Reviewer #2: Yes

**********

6. Review Comments to the Author

Please use the space provided to explain your answers to the questions above and, if applicable, provide comments about issues authors must address before this protocol can be accepted for publication. You may also include additional comments for the author, including concerns about research or publication ethics.

You may also provide optional suggestions and comments to authors that they might find helpful in planning their study.

(Please upload your review as an attachment if it exceeds 20,000 characters)

Reviewer #1: The manuscript presents a generally valid and relevant rationale for the proposed study. The research problem is clearly articulated and addresses an important and timely gap in the existing literature. The study appears well-positioned to contribute meaningfully to the academic knowledge base while also offering potential implications for policy and practice. The background section adequately describes the significance of the topic and situates the study within ongoing scholarly and policy discussions.

That said, the rationale could be further strengthened by more explicitly linking the research questions to an overarching conceptual or theoretical framework. While the problem statement is clear, the manuscript would benefit from a clearer articulation of the theoretical lens guiding the inquiry. Explicitly connecting the research questions to established frameworks would enhance conceptual coherence and help readers better understand how the study advances knowledge beyond descriptive or exploratory objectives. In particular, the authors should clarify how this work builds upon, diverges from, or fills limitations in prior studies. A more explicit statement of the expected contribution—whether methodological, theoretical, empirical, or policy-oriented—would further strengthen the justification for the study.

From a broader global health perspective, the framing of the research questions could be enriched by aligning them with established health promotion principles. For instance, the framework articulated in the Ottawa Charter for Health Promotion emphasizes building healthy public policy, creating supportive environments, strengthening community action, developing personal skills, and reorienting health services. If the proposed study relates to systems strengthening, governance, prevention, or population-level interventions, explicitly situating the research within one or more of these action areas would enhance theoretical grounding and global relevance. Integrating such a perspective would also strengthen the manuscript’s policy implications and demonstrate alignment with internationally recognized public health principles.

Regarding technical soundness, the protocol appears generally well designed and capable of producing meaningful findings. However, greater methodological specificity is needed to ensure transparency and minimize undisclosed analytical flexibility. The manuscript would benefit from clearer specification of primary and secondary outcomes, particularly if multiple endpoints are anticipated. Precise operational definitions of key variables and outcomes would help reduce ambiguity and improve reproducibility.

In addition, the analytic strategy requires more detailed elaboration. The authors should specify the statistical or analytical models to be employed, the assumptions underlying these models, and the rationale for selecting them. It would be helpful to clarify which analyses are confirmatory (hypothesis-testing) and which are exploratory in nature. If certain analytic decisions will depend on the characteristics of the collected data, this should be explicitly acknowledged, along with predefined criteria guiding those decisions. Where applicable, inclusion of a statistical power calculation or justification of sample size would further strengthen the protocol’s rigor. If power analysis is not feasible due to the nature of the study design, the authors should provide a clear explanation.

The manuscript should also more clearly describe how potential sources of bias will be minimized. This includes selection bias, measurement bias, confounding factors, and analytical bias. Strategies for handling missing data, addressing potential floor or ceiling effects, and conducting sensitivity analyses (if applicable) should be outlined in advance. Providing this level of detail will enhance the credibility of the proposed research and ensure that readers can assess the robustness of the methodology.

If the study evaluates policy, system-level, or population-based interventions, it would be beneficial for the authors to explain how contextual variables and social determinants of health will be accounted for in the analysis. Health outcomes and implementation processes are often shaped by socioeconomic, environmental, and governance-related factors. Acknowledging and planning for these influences would align the study more closely with systems-based and equity-oriented approaches to public health research.

In terms of feasibility and replicability, the methodology is described in reasonable detail; however, additional clarification is needed to ensure full reproducibility. The authors should provide detailed inclusion and exclusion criteria, where applicable, and clarify the sampling strategy and recruitment procedures. If specific instruments or tools will be used for data collection, these should be described comprehensively, including information on their validity and reliability where available. Timelines and procedural steps should be clearly outlined to allow another researcher to replicate the workflow.

If the study involves qualitative methods or mixed methods approaches, details regarding coding frameworks, inter-rater reliability procedures, and validation processes should be included. If it is a review-based protocol, the search strategy should be fully specified, including databases to be searched, search strings, date limits, language restrictions, screening procedures, and data extraction methods. Clarifying whether screening and extraction will be conducted independently by multiple reviewers, and how disagreements will be resolved, is important for ensuring transparency. Explicit quality control mechanisms should be described to demonstrate rigor.

Data availability is another area that requires further clarification. The manuscript should include a detailed Data Availability Statement that aligns with PLOS Data Policy. The authors should clearly indicate where datasets will be deposited upon completion of the study, whether in a recognized public repository or as supplementary materials. They should specify whether data will be available without restriction and how supporting materials—such as analytic code, extraction sheets, instruments, or protocols—will be shared. If any restrictions apply, such as those related to participant privacy or third-party data agreements, these should be transparently described. For studies involving human participants, anonymization procedures and data protection safeguards should be explicitly outlined. Full compliance with open data requirements should be clearly articulated prior to acceptance.

In terms of presentation and language, the manuscript is generally intelligible and logically structured. The overall flow is coherent, and the sections follow a clear progression. However, minor revisions would improve clarity and readability. Some sentences are lengthy and could be simplified for precision. Reducing repetition in the introduction would improve conciseness. Consistent terminology should be used throughout, and all acronyms should be defined at first use. Careful proofreading is recommended to correct minor typographical and grammatical issues. Since PLOS ONE does not copyedit accepted manuscripts, ensuring linguistic clarity prior to publication is essential.

Overall, this protocol addresses an important and policy-relevant topic with strong potential to contribute to the evidence base. The research is timely and has the capacity to inform both academic discourse and practical implementation. To strengthen the manuscript prior to acceptance, the authors should enhance the conceptual framework, provide more detailed methodological specifications, clarify confirmatory versus exploratory analyses, include power calculations where applicable, strengthen the Data Availability statement, and refine the manuscript for clarity and consistency.

From a broader public health standpoint, explicit integration of health promotion and systems-thinking approaches would further enhance the manuscript’s global relevance. Consideration of equity implications and potential differential impacts across population subgroups would strengthen its policy applicability. Addressing social determinants, multisectoral coordination, and governance dimensions—where relevant—would further align the study with contemporary global health priorities.

In conclusion, the protocol demonstrates substantial promise but requires moderate revisions to ensure methodological transparency, reproducibility, and conceptual robustness. With these improvements, the study has the potential to make a meaningful and valuable contribution to the field.

Reviewer #2: This protocol addresses an important and relevant topic, and the use of a realist review is appropriate for examining how educational strategies improve health literacy in adults with type 2 diabetes in low socioeconomic communities. The rationale is clear, the objectives are meaningful, and the manuscript shows good alignment with realist review principles and RAMESES guidance.

However, the protocol would benefit from revision before publication. The main limitation is insufficient methodological detail. The search strategy is described only broadly; the manuscript should provide a more reproducible search approach, including clearer operationalisation of search terms, database strategy, and iterative search procedures. In addition, the processes for study selection, appraisal, and data extraction need sharper detail, particularly how relevance, richness, and rigour will be judged consistently across reviewers.

The analytic section would also be strengthened by clarifying how ICAMO configurations will be developed from extracted data and how competing explanations will be handled. Finally, the manuscript would benefit from minor language and formatting revision for consistency. Overall, this is a worthwhile protocol with potential value, but further clarification is needed to improve transparency and reproducibility before acceptance.

**********

7. PLOS authors have the option to publish the peer review history of their article (what does this mean?). If published, this will include your full peer review and any attached files.

If you choose “no”, your identity will remain anonymous but your review may still be made public.

**Do you want your identity to be public for this peer review?**  For information about this choice, including consent withdrawal, please see our Privacy Policy.

Reviewer #1: No

Reviewer #2: **Yes:**  Mansuor A. Alanazi

**********

[NOTE: If reviewer comments were submitted as an attachment file, they will be attached to this email and accessible via the submission site. Please log into your account, locate the manuscript record, and check for the action link "View Attachments". If this link does not appear, there are no attachment files.]

To ensure your figures meet our technical requirements, please review our figure guidelines: https://journals.plos.org/plosone/s/figures

You may also use PLOS’s free figure tool, NAAS, to help you prepare publication quality figures: https://journals.plos.org/plosone/s/figures#loc-tools-for-figure-preparation.

NAAS will assess whether your figures meet our technical requirements by comparing each figure against our figure specifications.

Attachment Submitted filename: 1. PLOS ONE Reviewer Repor.docx..docx

# Author comment: Author response to Decision Letter 1

**DOI:** 10.1371/journal.pone.0352599.r002

7 Jun 2026

We thank the reviewers for the time and diligence in providing their feedback.

An itemized response is added as a separate file.

Attachment Submitted filename: Responses to Reviewers comments.docx

# Editor report: Decision Letter 1

**DOI:** 10.1371/journal.pone.0352599.r003

**Authors:** Marwan Al-Nimer

12 Jun 2026

Educational strategies to improve health literacy for people with type 2 diabetes in low socio-economic communities: A realist review protocol

PONE-D-25-49650R1

Dear Dr. Martin Heine,

We’re pleased to inform you that your manuscript has been judged scientifically suitable for publication and will be formally accepted for publication once it meets all outstanding technical requirements.

Within one week, you’ll receive an e-mail detailing the required amendments. When these have been addressed, you’ll receive a formal acceptance letter and your manuscript will be scheduled for publication.

An invoice will be generated when your article is formally accepted. Please note, if your institution has a publishing partnership with PLOS and your article meets the relevant criteria, all or part of your publication costs will be covered. Please make sure your user information is up-to-date by logging into Editorial Manager at Editorial Manager® and clicking the ‘Update My Information' link at the top of the page. For questions related to billing, please contact billing support.

If your institution or institutions have a press office, please notify them about your upcoming paper to help maximize its impact. If they’ll be preparing press materials, please inform our press team as soon as possible -- no later than 48 hours after receiving the formal acceptance. Your manuscript will remain under strict press embargo until 2 pm Eastern Time on the date of publication. For more information, please contact onepress@plos.org.

Kind regards,

Marwan Salih Al-Nimer, MD, PhD

Academic Editor

PLOS One

Additional Editor Comments (optional):

Reviewers' comments:

# Editor report: Acceptance letter

**DOI:** 10.1371/journal.pone.0352599.r004

**Authors:** Marwan Al-Nimer

PONE-D-25-49650R1

PLOS One

Dear Dr. Heine,

I'm pleased to inform you that your manuscript has been deemed suitable for publication in PLOS One. Congratulations! Your manuscript is now being handed over to our production team.

At this stage, our production department will prepare your paper for publication. This includes ensuring the following:

* All references, tables, and figures are properly cited

* All relevant supporting information is included in the manuscript submission,

* There are no issues that prevent the paper from being properly typeset

You will receive further instructions from the production team, including instructions on how to review your proof when it is ready. Please keep in mind that we are working through a large volume of accepted articles, so please give us a few days to review your paper and let you know the next and final steps.

Lastly, if your institution or institutions have a press office, please let them know about your upcoming paper now to help maximize its impact. If they'll be preparing press materials, please inform our press team within the next 48 hours. Your manuscript will remain under strict press embargo until 2 pm Eastern Time on the date of publication. For more information, please contact onepress@plos.org.

You will receive an invoice from PLOS for your publication fee after your manuscript has reached the completed accept phase. If you receive an email requesting payment before acceptance or for any other service, this may be a phishing scheme. Learn how to identify phishing emails and protect your accounts at https://explore.plos.org/phishing.

If we can help with anything else, please email us at customercare@plos.org.

Thank you for submitting your work to PLOS ONE and supporting open access.

Kind regards,

PLOS ONE Editorial Office Staff

on behalf of

Professor Marwan Salih Al-Nimer

Academic Editor

PLOS One
